# Nanoformulation Shows Cytotoxicity against Glioblastoma Cell Lines and Antiangiogenic Activity in Chicken Chorioallantoic Membrane

**DOI:** 10.3390/pharmaceutics13060862

**Published:** 2021-06-11

**Authors:** Danieli Rosane Dallemole, Thatiana Terroso, Aline de Cristo Soares Alves, Juliete Nathali Scholl, Giovana Ravizzoni Onzi, Rodrigo Cé, Karina Paese, Ana Maria Oliveira Battastini, Silvia Stanisçuaski Guterres, Fabrício Figueiró, Adriana Raffin Pohlmann

**Affiliations:** 1 Graduate Program in Pharmaceutical Science, Faculty of Pharmacy, Federal University of Rio Grande do Sul, Av. Ipiranga, 2752, Porto Alegre, RS 90610-000, Brazil; danieli.dallemole@ufrgs.br (D.R.D.); thatiterroso@gmail.com (T.T.); alves.alinecs@yahoo.com.br (A.d.C.S.A.); gioonzi@gmail.com (G.R.O.); rodrigoce_@gmail.com (R.C.); karina.paese@ufrgs.br (K.P.); silvia.guterres@ufrgs.br (S.S.G.); 2 Graduate Program in Biological Sciences: Biochemistry, Institute of Health Sciences, Federal University of Rio Grande do Sul, Ramiro Barcelos Street, 2600, Porto Alegre, RS 90035-003, Brazil; juliete.scholl@gmail.com (J.N.S.); abattastini@gmail.com (A.M.O.B.); 3 Department of Production and Control of Medicines, Federal University of Rio Grande do Sul, Av. Ipiranga, 2752, Porto Alegre, RS 90610-000, Brazil; 4 Department of Biochemistry, Institute of Health Sciences, Federal University of Rio Grande do Sul, Ramiro Barcelos Street, 2600, Porto Alegre, RS 90035-003, Brazil

**Keywords:** glioblastoma, multi-drug delivery systems, lipid-core nanocapsules, surface functionalization, CAM assay

## Abstract

Glioblastoma (GB) is a histological and genetically heterogeneous brain tumor that is highly proliferative and vascularized. The prognosis is poor with currently available treatment. In this study, we evaluated the cytotoxicity and antiangiogenic activity of doxorubicin-loaded-chitosan-coated-arginylglycylaspartic acid-functionalized-poly(ε-caprolactone)-alpha bisabolol-LNC (AB-DOX-LNC-L-C-RGD). The nanoformulation was prepared by self-assembling followed by interfacial reactions, physicochemically characterized and evaluated in vitro against GB cell lines (U87MG and U138MG) and in vivo using the chicken chorioallantoic membrane assay (CAM). Spherical shape nanocapsules had a hydrodynamic mean diameter of 138 nm, zeta potential of +13.4 mV, doxorubicin encapsulation of 65%, and RGD conjugation of 92%. After 24 h of treatment (U87MG and U138MG), the median inhibition concentrations (IC_50_) were 520 and 490 nmol L^−1^ doxorubicin-equivalent concentrations, respectively. The treatment induced antiproliferative activity with S-phase cell-cycle arrest and apoptosis in the GB cells. Furthermore, after 48 h of exposure, evaluation of antiangiogenic activity (CAM) showed that the relative vessel growth following treatment with the nanocapsules was 5.4 times lower than that with the control treatment. The results support the therapeutic potential of the nanoformulation against GB and, thereby, pave the way for future preclinical studies.

## 1. Introduction

Glioblastoma (GB) is the most common and aggressive tumor in the central nervous system. The median overall survival of patients with GB after diagnosis is approximately 15 months [1]. The current standard treatment consists of surgical resection, radiotherapy, and chemotherapy (primarily temozolomide) [2]. However, this tumor is characterized by histopathological and genetic heterogeneity, high proliferation rate, and invasiveness of adjacent tissues. Consequently, complete resection is difficult, causing tumor recurrence [3]. Additionally, the blood–brain barrier provides an obstacle for the delivery of drugs to the central nervous system [4,5].

Furthermore, GB growth, maintenance, and progression are supported by angiogenesis. Angiogenesis is the development of new blood vessels from preexisting ones, induced by hypoxia and the expression of proangiogenic factors such as vascular endothelial growth factor in the tumor microenvironment. The new tumor blood vessels are formed quickly, therefore, the vessel architecture is disorganized and sinuous with enlarged gaps between the endothelial cells. This aspect, associated with poor lymphatic drainage, enhances the permeability and retention effect in the tumor [6,7,8].

Another characteristic of GB is the overexpression of transmembrane cell-surface receptors such as integrin αvβ3 by tumor and endothelial cells [9,10]. Integrins regulate diverse functions including cell adhesion to the extracellular matrix, migration, proliferation, tumor invasion, and tumor angiogenesis [11]. Under these circumstances, GB treatment is a challenge.

Recent studies have demonstrated the antineoplastic effect of doxorubicin against GB cell lines and xenograft models [12,13]. Doxorubicin is an anthracycline antibiotic that is widely used in the clinical treatment of various solid tumors and leukemia. In parallel, alpha-bisabolol, a sesquiterpene alcohol, exhibits pharmacological properties such as anti-inflammatory, antibiotic, gastroprotective, antiangiogenic, and antitumor activities. In tumor cells including GB cells, alpha-bisabolol induces the loss of plasma and mitochondrial membrane integrity, decreasing cell viability [14,15].

In the last few decades, nanocarriers have been investigated as an approach for overcoming the limitations of GB treatments [5,16]. Due to their nanometric size, nanostructured systems can diffuse from the vessels into the tumor environment (passive targeting), and surface-functionalized nanocarriers with specific ligands facilitate particle internalization by cells at the target site (active targeting) [4,17]. Another strategy used to decrease tumor development is inhibition of the tumor angiogenesis process [18].

Our research group developed lipid-core nanocapsule (LNC) formulations [19], which enables the encapsulation of different lipophilic drugs showing to be promising for the treatment of various cancers such as breast cancer [20], cervical carcinoma [21], and leukemia [22]. Moreover, we demonstrated that LNCs permeate through the blood–brain barrier, increasing the intratumoral bioavailability of drugs and reducing the tumor size in an in vivo model of GB [23,24].

Surface functionalized LNCs can be produced by coating with lecithin, chitosan, and polysorbate 80, followed by interfacial reactions with metal ions (Fe^2+^, Zn^2+^, or Au^3+^), which allows the complexation with different ligands: anti-LDL(−)-single-chain variable antibody fragment for inhibiting atheroma progression [25,26], laronidase for treating mucopolysaccharidosis type I [27], methotrexate showing enhanced and selective antiproliferative effects against human breast cancer cells [20], bevacizumab demonstrating in vitro cytotoxicity against the C6 cell line and in vivo antiangiogenic activity [28], and arginylglycylaspartic acid (RGD) as a ligand in doxorubicin-loaded nanocapsules [29]. In this last study, Pareto charts indicated that breast cancer (MCF-7) and glioma (U87MG) cell viabilities were primarily affected by the drug concentration and treatment duration and that RGD-surface functionalization is an important factor in reducing U87MG cell viability, compared with the viability in MCF-7 cells, due to the overexpression of αvβ3 integrin in glioma cells.

RGD-peptides can be used as integrin antagonists and nanocarrier ligands for mediation of therapeutic and theranostic targeting [11,30]. Furthermore, the recognition of RGD-peptides by integrins can promote cell detachment and, consequently, cell death [31]. On the basis of this phenomenon, RGD peptides have been used to target dendrimers, polymeric micelles and nanoparticles, liposomes, and inorganic nanocarriers to the tumor environment and tumor blood vessels [30,32].

One of the in vivo models used in studies of angiogenesis and inhibition of vessel growth is the chicken chorioallantoic membrane (CAM). CAM is an extra-embryonic membrane that becomes highly vascularized during embryonic development. This model has the advantage of easy handling and the visualization of blood vessels, it is an inexpensive assay, and can be used as an alternative model to the use of other experimental models [33,34].

Considering these reports, our objective was to develop and evaluate doxorubicin-loaded-chitosan-coated-arginylglycylaspartic acid-functionalized-poly(ε-caprolactone)-alpha bisabolol-LNC (AB-DOX-LNC-L-C-RGD) as an antitumor agent. In this study, we demonstrate that AB-DOX-LNC-L-C-RGD reduces the viability of U87MG and U138MG (GB cell lines) through programmed cell death, causing S-phase cell cycle arrest, even for cells resistant to temozolomide. In addition, using the in vivo CAM model, we showed that this nanoformulation is capable of inhibiting angiogenesis.

## 2. Materials and Methods

### 2.1. Materials

RGD (A8052), chitosan low molecular weight, dimethyl sulfoxide (DMSO), doxorubicin hydrochloride (D1515), poly(ε-caprolactone) (PCL 10, Mn 10,000 g mol^−1^, poly(ε-caprolactone) (PCL 80, Mn 80,000 g mol^−1^), polysorbate 80 (Tween^®^ 80), QuantiPRO™ BCA assay kit, sorbitan monostearate (Span^®^ 60), temozolomide (TMZ, T2577), zinc acetate, and 3-(4,5-dimethylthiazol-2yl)-2,5-diphenyltetrazolium bromide (MTT) were purchased from Sigma Aldrich, Saint Louis, MO, USA. Alpha-bisabolol was supplied by Fagron, São Paulo, Brazil. Lipoid^®^ S75 was obtained from Lipoid, Ludwigshafen, Germany. Dulbecco’s modified Eagle’s medium (DMEM), fetal bovine serum (FBS), penicillin/streptomycin, and trypsin/EDTA were supplied by Gibco, Carlsbad, CA, USA. The FITC Annexin V Apoptosis Detection Kit (annexin V-FITC/propidium iodide; PI) was obtained from BD Biosciences, San Jose, CA, USA. All solvents used were of analytical grade.

### 2.2. Preparation of the Surface-Functionalized Lipid-Core Nanocapsules

All formulations were prepared by interfacial deposition of preformed polymer followed by surface coating and functionalization, as previously reported [29,35,36]. To obtain doxorubicin-loaded-poly(ε-caprolactone)-alpha bisabolol-LNC (AB-DOX-LNC-L), 0.1 g of a mixture of PLC 80 and PCL 10 (9:1, *w*/*w*) was dissolved in acetone (25 mL) (organic phase I, OP-I). In parallel, we prepared organic phase II (OP-II) by adding 0.04 g of sorbitan monostearate, 0.16 g of alpha-bisabolol, 1 mg of doxorubicin hydrochloride, and triethylamine (14.5 μL; used to neutralize doxorubicin) in ethanol (3 mL). Organic phase III (OP-III) was prepared with 0.09 g of Lipoid^®^ S75 dissolved in ethanol (4 mL). All organic phases were maintained under magnetic stirring at 40 °C. Organic phases (OP-I, OP-II, and OP-III) were mixed. The resultant solution was injected using a funnel (50 mL, Eppendorf Combitips advanced^®^, Eppendorf, São Paulo, Brazil), under magnetic stirring, into an aqueous phase containing 0.08 g of polysorbate 80 and ultrapure water (50 mL). A translucent solution was formed instantaneously. After 10 min, the organic solvents and part of the water were removed under reduced pressure (rotator evaporator Buchi, Flawil, Switzerland) at 40 °C to concentrate the solution until a volume of 9 mL was reached. The final volume was adjusted with ultrapure water to 10 mL in a volumetric flask. Poly(ε-caprolactone)-alpha bisabolol-LNC (AB-LNC-L) was prepared without doxorubicin hydrochloride using the method described above for comparative purposes.

Subsequently, a chitosan aqueous solution (0.7%, *w*/*v*) containing acetic acid (1%, *v*/*v*) was prepared. The solution was filtered (0.45 μm, Merck Millipore^®^, Burlington, MA, USA) and added dropwise (1 mL) into AB-DOX-LNC-L (9 mL) under magnetic stirring for 2 h, which resulted in the formation of doxorubicin-loaded-chitosan-coated-poly(ε-caprolactone)-alpha bisabolol-LNC (AB-DOX-LNC-L-C). Similarly, AB-LNC-L was reacted with chitosan, generating chitosan-coated-poly(ε-caprolactone)-alpha bisabolol-LNC (AB-LNC-L-C).

Surface functionalization was performed by reacting 975 μL of AB-DOX-LNC-L-C with 25 μL of 1 mg mL^−1^ of zinc acetate aqueous solution under magnetic stirring. After 1 min, 351.21 μL of this mixture was transferred to a new flask containing 648.79 μL of 450 μg mL^−1^ RGD aqueous solution. The reaction mixture was stirred for 10 min at 25 °C to produce AB-DOX-LNC-L-C-RGD. Similarly, AB-LNC-L-C was reacted with zinc acetate and RGD, which resulted in the chitosan-coated-arginylglycylaspartic acid-functionalized-poly(ε-caprolactone)-alpha bisabolol-LNC formulation (AB-LNC-L-C-RGD).

It is important to mention that the alpha-bisabolol amount used in this study was selected according to a previous study that determined the ideal proportion between sorbitan monostearate, oil, and polymer (1:4.1:2.6, *w*/*w*/*w*) [19], while doxorubicin and RGD concentrations were based on the antitumor effect previously observed for RGD-functionalized doxorubicin-nanocapsules [29].

### 2.3. Physicochemical Characterization

The hydrodynamic mean diameter of the particles (z-average diameter) as well as the polydispersity index (PDI) were determined using dynamic light scattering (DLS) (ZetaSizer Nano ZS, Malvern, UK). In this method, a sample of each formulation was diluted 500× in previously filtered ultrapure water (0.45 μm, Merck Millipore^®^). The analysis was performed at 25 °C, and light scattering was detected at an angle of 173°.

The zeta potential was calculated after determining the electrophoretic mobility (ZetaSizer Nano ZS, Malvern, UK). A sample of each formulation was diluted (500×) in a previously filtered aqueous solution of 10 mmol L^−1^ sodium chloride.

Nanoparticle tracking analysis (NTA) (NanoSight LM10, Nanosight, UK) was performed to determine the particle number density (particles mL^−1^) (PND) and mean diameter based on the number of particles. The formulations were diluted (1:10,000, *v*/*v*) in previously filtered (0.45 μm, Merck Millipore^®^) ultrapure water and inserted into the sample holder chamber. Six videos were recorded for each sample (triplicate batches, *n* = 3), at room temperature, using shutter and manual gain adjustments. Each video was captured over 10 s. PND was converted to micromolar concentrations of nanocapsules per liter using Avogadro’s number (6.023 × 10^23^) [29].

The pH values were determined via direct insertion of the probe into the formulation (without previous preparation) at room temperature using a potentiometer (UB-10, Denver Instruments, New York, NY, USA) that was calibrated with pH 4.0 and 7.0 buffer solutions. Considering the scale of production selected in this study for the preparation of the RGD-surface-functionalized formulations, such samples were analyzed using pH indicator strips (Merck Millipore^®^).

The RGD complexation efficiency was evaluated via colorimetric analysis using the QuantiPRO™ BCA Kit (Sigma Aldrich, Saint Louis, MI, USA). Standard curves were prepared using dilutions of RGD aqueous solution at 3–27 μg mL^−1^. Absorbance was read at 562 nm using a microplate reader (Spectramax, Molecular Devices, San Jose, CA, USA). The RGD aqueous solution at 450 μg mL^−1^ was experimentally quantified before it was used as a reactant for the surface-functionalization step of the synthesis. Subsequently, 400 μL of the RGD-surface-functionalized formulation was added to a 30 kDa filter unit (Merck Millipore^®^) and centrifuged at 1844× *g* for 5 min to isolate the non-complexed fraction of RGD [29]. The RGD complexation efficiency (CE%) was determined according to Equation (1).

(1) $$\mathrm{CE}\%=\frac{\left(RGDt-RGDf\right)}{RGDt}\times 100,$$

where *RGDt* is the total *RGD* content and *RGDf* is the *RGD* soluble concentration in the ultrafiltrate.

The alpha-bisabolol content was quantified by liquid chromatography (HPLC, Shimadzu Corporation, Kyoto, Japan) on the basis of a previously described method with modifications [37]. For this quantification, an aliquot of 20 μL of formulation was added to a volumetric flask containing acetonitrile (10 mL), filtered, and analyzed by HPLC. The stationary phase consisted of a C18 column (Luna 5 μm, Phenomenex, 150 × 4.60 mm). Acetonitrile:water (85:15, *v*/*v*) was used as the mobile phase. The flow rate was 1.0 mL min^−1^, the injection volume was 40 μL, and the drug was detected at 207 nm. The encapsulation efficiency (EE%) was determined after ultrafiltration–centrifugation based on a previously reported methodology [37], in which 400 μL of the formulation was filtered through a 10 kDa filter unit (Merck Millipore^®^) and subjected to centrifugation at 13,500× *g* for 10 min. EE% was determined according to Equation (2).

(2) $$\mathrm{EE}\;\%=\frac{\left(Ct-Cf\right)}{Ct}\times 100,$$

where *Ct* is the total content and *Cf* is the concentration of the non-encapsulated drug in the ultrafiltrate.

Additionally, the doxorubicin content was determined by HPLC using an RP-18 mm column (150 mm × 4.6 mm × 5 μm, ODS2 Waters Spherisorb®, Waters Corporation, Milford, MA, USA). The mobile phase consisted of acetonitrile:water 50:50, *v*/*v*) with an apparent pH of 2.65, adjusted with aqueous solution of trifluoroacetic acid (25% *v*/*v*). First, 500 μL of each formulation was added to 5 mL of the mobile phase [29]. The mixture was stirred for 15 min, filtered (0.45 μm, Merck Millipore^®^), and analyzed by HPLC. The flow rate was 1.0 mL min^−1^, the injection volume was 50 μL, and the drug was detected at 254 nm. EE% was determined according to Equation (2), following ultrafiltration through a 10 kDa filter unit (Merck Millipore^®^) and centrifugation at 1844× *g* for 15 min. All analyses were performed in triplicate batches.

The AB-DOX-LNC-L-C-RGD formulation was analyzed by transmission electron microscopy (TEM; JEM 1200 EXII TEM, JEOL) at the Microscopy and Microanalysis Center of the Federal University of Rio Grande do Sul (CMM-UFRGS).

### 2.4. Biological Assays

#### 2.4.1. Cell Culture Conditions

GB cell lines (U87MG and U138MG), human hepatocellular carcinoma-HepG2, and normal cell lines (human keratinocytes, HaCaT; human lung fibroblasts, MRC-5) were obtained from American Type Culture Collection (ATCC, Rockville, MD, USA) and maintained in DMEM supplemented with FBS (10% *v*/*v*, pH 7.4) and penicillin/streptomycin (0.5 U mL^−1^) at 37 °C in a 5% CO_2_ atmosphere and 95% relative humidity.

#### 2.4.2. Preparation of the Stock and Working Solution for the In Vitro Assays

Temozolomide (TMZ) was solubilized in DMSO at 100 mmol L^−1^ (solution I). Subsequently, solution I was diluted with DMSO to prepare stock solutions at TMZ concentrations of 50 mmol L^−1^, 40 mmol L^−1^, 30 mmol L^−1^, 20 mmol L^−1^, and 10 mmol L^−1^. Subsequently, the stock solutions were diluted in DMEM to obtain working solutions at TMZ concentrations of 100–500 μmol L^−1^. The final concentration of DMSO in the TMZ treatment solutions was 1%.

Additionally, working solutions containing the formulation AB-DOX-LNC-L-C-RGD were prepared; thus, different volumes of AB-DOX-LNC-L-C-RGD were diluted in DMEM to obtain doxorubicin final concentrations of 0.01–1.0 μmol L^−1^ (information on the corresponding concentrations of nanocapsules, alpha-bisabolol, and RGD is described in Supplementary Materials Table S1).

All experiments were conducted using three different negative controls: untreated cells (control 1), ultrapure water (control 2), and 1% DMSO solution (control 3). Control 2 was prepared by adding a volume of ultrapure water in DMEM equivalent to the highest volume of formulation used in the in vitro assays. Control 3 was prepared by diluting DMSO in DMEM at 1%.

#### 2.4.3. Cellular Uptake of AB-DOX-LNC-L-C-RGD

U87MG and U138MG cells were cultivated on 6-well culture plates until they reached semi-confluence. Subsequently, the medium was removed and replaced with 1 mL of working solution containing AB-DOX-LNC-L-C-RGD (0.01–1.0 μmol L^−1^) (prepared as described in “Preparation of the stock and working solution for the in vitro assays”), control 1 (untreated cells), or control 2 (ultrapure water), and incubated for 6 h. Next, the cells and the medium containing the working solutions of AB-DOX-LNC-L-C-RGD were centrifuged at 400× *g* for 6 min, washed with phosphate buffer saline (PBS), centrifuged, and suspended in PBS. The cellular uptake of the drug was estimated using flow cytometry (FACSCalibur, BD Biosciences, USA); the intrinsic fluorescence of doxorubicin was measured. The data were obtained from three independent experiments (*n* = 3) and analyzed using the FlowJo^®^ software (BD biosciences, USA).

#### 2.4.4. Cell Viability Assay

Cell viability was evaluated using the MTT assay. GB, hepatocellular carcinoma, and normal cells were seeded in 96-well plates and allowed to grow until they reached semi-confluence. Subsequently, the medium was removed from each well and replaced with 100 μL of one of the following working solutions: AB-DOX-LNC-L-C-RGD (0.01–1.0 μmol L^−1^) (Supplementary Materials Table S1), TMZ (100–500 μmol L^−1^), control 2 (ultrapure water), or control 3 (1% DMSO solution). After 24 h, we removed the treatment solutions, added 100 μL of MTT solution (5 mg mL^−1^) to each well, and incubated the cells at 37 °C for 2 h. The formazan crystals formed were dissolved in DMSO (100 μL) and absorbance was measured at 570 nm and 630 nm on a plate reader equipment (Spectramax, Molecular Devices, USA). The results were expressed as the percentage of viable cells relative to the untreated cells (control 1), which corresponded to 100% of cell viability. The data were obtained from three independent experiments (*n* = 3).

#### 2.4.5. Cell Cycle Analysis and Apoptosis Assay

The GB cells were seeded on 6-well culture plates and cultivated until semi-confluence. Subsequently, the medium was removed and replaced with 1 mL of a working solution containing AB-DOX-LNC-L-C-RGD at 0.5 μmol L^−1^ doxorubicin-equivalent concentration; a TMZ solution (500 μmol L^−1^); or controls 1, 2, or 3. After 24 h, the medium containing the treatments and cells were centrifuged at 400× *g* for 6 min. Subsequently, the cells were washed with PBS, centrifuged, and suspended in staining solution (0.5 mM Tris-HCl at pH 7.6, 3.5 mM trisodium citrate, 0.1% nonidet 40 (*v*/*v*), 100 μg mL^−1^ RNase, and 50 μg mL^−1^ PI). To perform the apoptosis assay, after the centrifugation step, the cells were washed twice with PBS, centrifuged, and suspended in buffer containing Annexin V for 15 min. After incubation time, the cell cycle or apoptosis was analyzed using a flow cytometer FACSCalibur (BD Bioscience, San Jose, CA, USA). The data were obtained from three independent experiments (*n* = 3) and analyzed using the FlowJo^®^ 7.6.5 software.

#### 2.4.6. Antiangiogenic Assay on Chicken Embryo Chorioallantoic Membrane

The CAM assay was previously approved by the Animal Ethical Committee of the Universidade Federal do Rio Grande do Sul (protocol no. 33993). Fertilized chicken eggs (*Gallus domesticus*) were cleaned with 70% (*v*/*v*) alcohol and subsequently incubated at 37 °C, and a relative humidity of 80% in a climatic chamber until the sixth day of development. Following this initial incubation period, a small opening was made in the air chamber, the inner shell membrane was removed, and the CAM was photographed (initial assay time: t0). To maintain the covered area of the chorioallantoic membrane constant to guarantee the viability of the eggs within the entire period of the experiment, we selected 100 μL as the applied volume for all treatments and control. A physiological solution (0.9% NaCl, *w*/*v*) was used as the negative control. Thus, 49 eggs were randomly divided into 10 groups (*n* = 4 or 5) and treated with negative control or three different concentrations of nanocapsules for each formulation: AB-LNC-L-C (2.21 × 10^−3^, 4.42 × 10^−3^, and 8.85 × 10^−3^ μmol L^−1^); AB-LNC-L-C-RGD (2.03 × 10^−3^, 4.06 × 10^−3^, and 8.12 × 10^−3^ μmol L^−1^), AB-DOX-LNC-L-C-RGD (2.04 × 10^−3^, 4.08 × 10^−3^, and 8.17 × 10^−3^ μmol L^−1^) (Supplementary Materials Table S2). To obtain these concentrations, different volumes of formulations were diluted in 0.9% NaCl solution. Subsequently, the eggs were sealed with cleaned parafilm to decrease microbial contamination and prevent embryo dehydration and incubated for 24 h. Next, the parafilm was removed and new images were recorded, characterizing the treatment time of 24 h (t24). The eggs were sealed and incubated for an additional 24 h and the imaging process was repeated, resulting in 48 h of total treatment exposure on CAM (t48).

The photographs were transformed into grayscale using the software Adobe^®^ Photoshop^®^ CS2 version 9.0. Subsequently, the images were analyzed to determine the mean pixel value using the software ImageJ, on the basis of the method established by Alves et al. with modifications [28]. To perform this analysis, circular areas of 282,792 pixels^2^ were selected in the images before (t0) and after 24 h (t24) and 48 h (t48) of treatment for determination of the mean pixel value for each image. The scale of pixel values ranges from 0 to 255, where 0 refers to black and 255 to white. Thus, when angiogenesis increases, the mean pixel value decreases (closer to 0), and when angiogenesis decreases, as reflected by the inhibition of vessel growth, the mean pixel value increases (closer to 255). The mean pixel value was used to calculate the relative vessel growth (RVG) (Equation (3)).

(3) $$\mathrm{RVG}\;\left(\%\right)=\frac{\left(Pi-Pf\right)}{Pf}\times 100,$$

where *Pi* is the mean pixel value at the initial time point (t0) and *Pf* is the mean pixel value at the final time point (t24 or t48) of analysis. Because the observed area was constant in all photographs, this variable was omitted from the equation.

### 2.5. Statistical Analysis

Statistical analysis was performed using the GraphPad Prism 5.0 software (GraphPad Software Inc., La Jolla, CA, USA). The data were expressed as mean values and standard deviations (SD) for the physicochemical assays. For biological assays, the data were expressed as mean values and standard error of the mean (SEM). Differences between the experimental groups were compared using analysis of variance (ANOVA) followed by the Dunnett or Tukey (for multiple comparisons) post-hoc test and considered significant when *p* < 0.05. IC_50_ values were calculated using linear regression analysis.

## 3. Results

### 3.1. Nanoformulations

Macroscopically, the formulations prepared without doxorubicin were homogeneous white opalescent liquids, while the formulations containing the antineoplastic drug exhibited a homogeneous whitish-pink aspect. DLS analysis showed unimodal curves with narrow size distributions (PDI < 0.2) (Supplementary Materials Figure S1). The hydrodynamic mean diameters (z-average diameter, calculated using the method of Cumulants) varied from 138 to 147 nm (Table 1). Comparing the formulations prepared with and without doxorubicin, the drug encapsulation and surface functionalization did not significantly change the z-average diameters (*p* > 0.05, ANOVA, Tukey).

**Figure 1:**
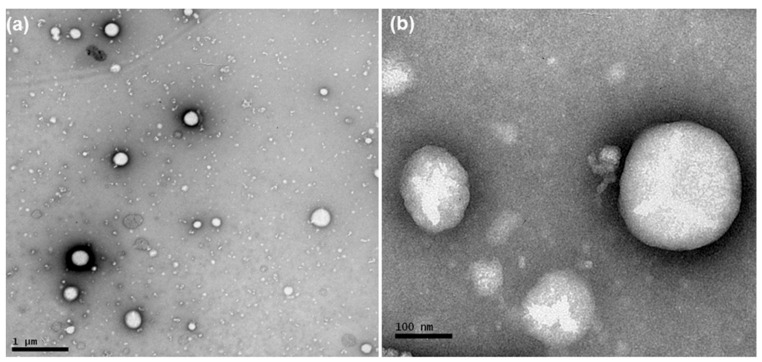
Photomicrographs obtained by transmission electron microscopy of AB-DOX-LNC-L-C-RGD: (**a**) bar = 1 μm and (**b**) bar = 100 nm.

According to NTA analysis, the mean diameter of the particles and PND were 136–170 nm and 4.89 × 10^12^–2.44 × 10^13^, respectively (Table 1). Photomicrographs of AB-DOX-LNC-L-C-RGD nanocapsules, as obtained using TEM, showed spherical structures (Figure 1). The diameters of these structures were in the range of diameter distributions determined by DLS and NTA.

The zeta potential of the uncoated formulation was negative and after coating with chitosan, it was inverted to positive values. The RGD-surface-functionalized nanocapsules maintained positive zeta potential values (Table 1). AB-LNC-L and AB-DOX-LNC-L exhibited pH values of 5.79 and 7.30, respectively. The coated and functionalized formulations showed pH values close to 4.

The alpha-bisabolol content was close to the total drug added in formulation with encapsulation efficiency close to 100%. The total content of RGD was 288.93 μg mL^−1^, leading to RGD complexation of 92.21 ± 0.1%. The total content of doxorubicin was 94.88 ± 2.61 μg mL^−1^ (AB-DOX-LNC-L), and the encapsulation efficiency ranged from 98.07 ± 0.71% (AB-DOX-LNC-L) to 64.32 ± 7.15% (AB-DOX-LNC-L-C) (Table 2). Considering the PND, the encapsulation efficiencies of alpha-bisabolol and doxorubicin as well as the complexation efficiency of RGD, one single AB-DOX-LNC-L-C-RGD nanocapsule contained 4.9 × 10^6^ molecules of alpha-bisabolol, 4.5 × 10^3^ molecules of doxorubicin distributed in the nucleus and polymeric wall, and 9.4 × 10^4^ molecules of RGD on the surface.

### 3.2. Cellular Uptake of AB-DOX-LNC-L-C-RGD

The detection of doxorubicin intrinsic fluorescence by flow cytometry demonstrated that the drug internalization occurred in a concentration-dependent manner in both GB cells. In the highest applied concentration (1.0 μmol L^−1^), the percentage of cells considered as doxorubicin-positive was greater than 80% (Supplementary Materials Figure S2).

### 3.3. Antitumor Effects of AB-DOX-LNC-L-C-RGD

#### 3.3.1. Cell Viability

We investigated the effect of the AB-DOX-LNC-L-C-RGD formulation on the viability of GB cells (U87MG and U138). The nanocapsule concentrations in this study varied from 1.56 × 10^−6^ to 1.57 × 10^−4^ μmol L^−1^ (Supplementary Materials Table S1). Additionally, we evaluated the cytotoxicity of TMZ, the primary drug choice for GB treatment, for a comparative analysis.

**Figure 2:**
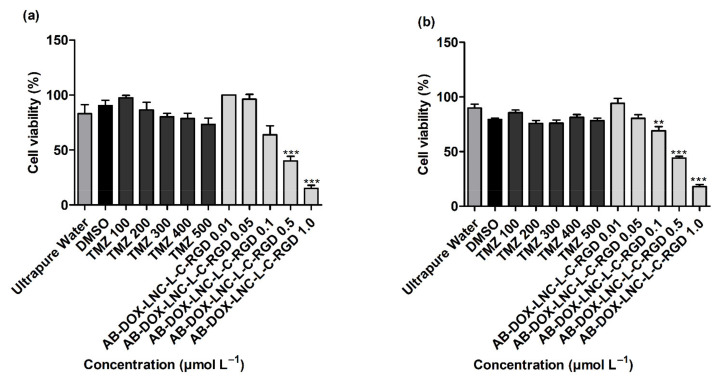
Cell viability evaluation using the MTT assay after 24 h of treatment on (**a**) U87MG cell line, IC_50_ = 0.52 ± 0.07 μmol L^−1^ (doxorubicin-equivalent) and (**b**) U138 cell line, IC_50_ 0.49 ± 0.03 μmol L^−1^ (doxorubicin-equivalent). Ultrapure water (control 2), DMSO (control 3). Data are expressed as the mean ± SEM. (*n* = 3) ** represents statistical difference with respect to the control (ultrapure water) (*p* < 0.01). *** represent the statistical difference with respect to the control (ultrapure water) (*p* < 0.001) (ANOVA, Dunnett).

As displayed in Figure 2a,b, more than 80% of the GB cells were viable after exposure to controls 2 and 3 (ultrapure water and DMSO 1%). TMZ treatments (100–500 μmol L^−1^) demonstrated no significant reduction in cell viability compared to DMSO (control 3) in both cell lines evaluated (*p* > 0.05). In contrast, treatment with AB-DOX-LNC-L-C-RGD at doxorubicin-equivalent concentrations of 0.5 and 1.0 μmol L^−1^ significantly reduced cell viability in U87MG (*p* < 0.001) (40.10 ± 4.06 and 15.23 ± 2.76% of cells remained viable, respectively) (Figure 2a). Similarly, AB-DOX-LNC-L-C-RGD exhibited cytotoxicity at doxorubicin-equivalent concentrations of 0.1, 0.5, and 1.0 μmol L^−1^ in U138MG (69.00 ± 3.80, 44.10 ± 1.51, and 17.93 ± 1.83% of cells remained viable, respectively) (Figure 2b). The calculated IC_50_ was 0.52 ± 0.07 and 0.49 ± 0.03 μmol L^−1^ (doxorubicin-equivalent concentrations) in U87MG and U138MG, respectively (Figure 2a,b) (optical microscopy cell images after treatment; Supplementary Materials Figure S3 and Figure S4). In parallel, we evaluated the formulation against the non-tumor cell lines HaCaT and MRC-5 and HepG2 cells. After 24 h of treatment, the formulation reduced the cell viability at all concentrations evaluated in HaCaT and MRC-5 (Supplementary Materials Figure S5). Cytotoxicity of AB-DOX-LNC-L-C-RGD in HepG2 was observed at 0.1, 0.5, and 1.0 μmol L^−1^ doxorubicin-equivalent concentration.

#### 3.3.2. Cell Cycle Analysis and Apoptosis

**Figure 3:**
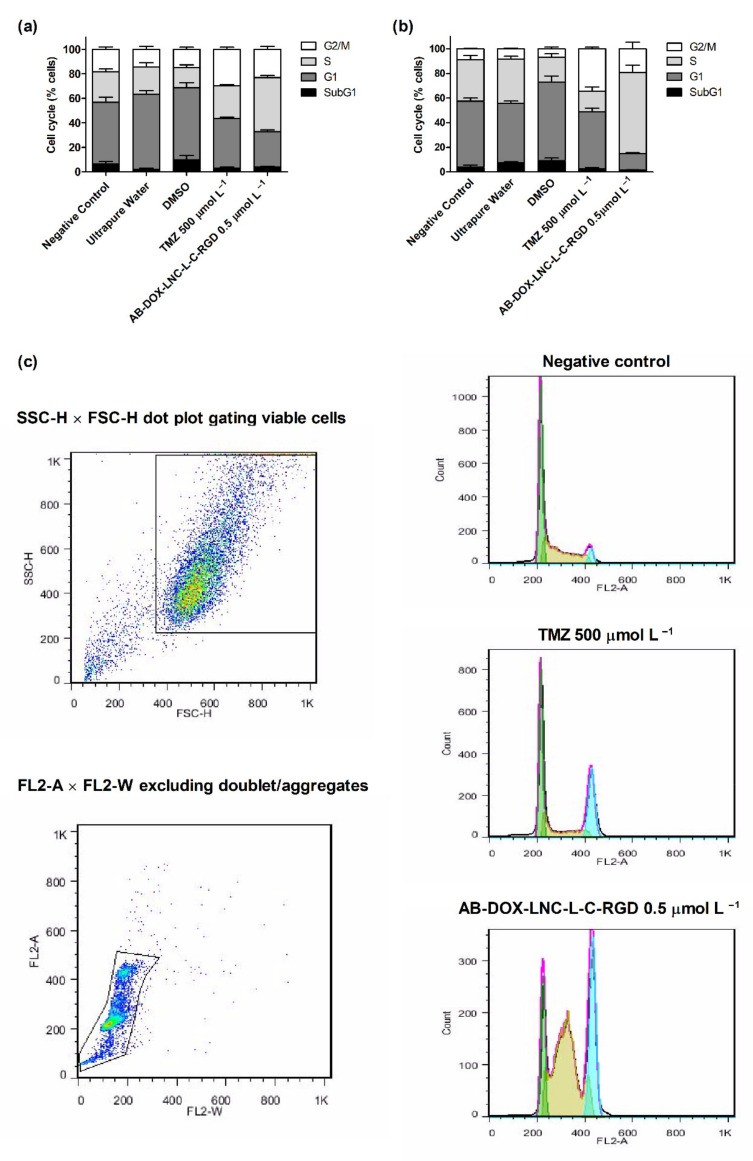
Cell cycle analysis using flow cytometry after 24 h of treatment for (**a**) U87MG cell line, (**b**) U138MG cell line, and (**c**) cell cycle representative histograms for U138MG cell line. Data are expressed as the mean ± SEM (*n* = 3). Negative control (untreated cells-control 1), ultrapure water (control 2), DMSO (control 3).

Cell cycle analysis was performed to evaluate the antiproliferative effect of the formulation. As shown in Figure 3a,b, exposure to TMZ resulted in G2/M cell cycle arrest. The cell population in this cycle cell phase was 29.79 ± 1.60% and 34.37 ± 1.26% for U87MG and U138MG, respectively, while after DMSO (control 3) exposure, the G2/M cell population was 14.93 ± 1.75% and 6.84 ± 1.43%, respectively. In contrast, AB-DOX-LNC-L-C-RGD promoted an increase in the population of cells in the S phase in both cell lines; however, this increase was more apparent in U138MG (see representative histograms in Figure 3c). In U87MG, the proportion of cells in the S phase increased to 44.03 ± 1.29% compared to 22.39 ± 3.63% for ultrapure water (control 2). The cell population in G1 decreased from 61.08 ± 3.20% (control 2) to 28.90 ± 1.40%, the G2/M and SubG1 populations increased from 14.52 ± 2.54% and 2.00 ± 0.96% after control 2 exposition to 23.16 ± 2.47% and 3.91 ± 0.68% after AB-DOX-LNC-L-C-RGD treatment (Figure 3a). A similar behavior was observed in U138MG culture, where the proportion of cells changed from 48.40 ± 1.89% (G1), 35.78 ± 2.27% (S), and 8.46 ± 0.53% (G2/M) for control 2 (ultrapure water) to 13.25 ± 0.78% (G1), 65.79 ± 5.99% (S), and 19.39 ± 5.32% (G2/M) after treatment with AB-DOX-LNC-L-C-RGD (Figure 3b). However, in the U138MG cell line, the SubG1 changed from 7.35 ± 0.81% (control 2) to 1.56 ± 0.21% after AB-DOX-LNC-L-C-RGD treatment.

**Figure 4:**
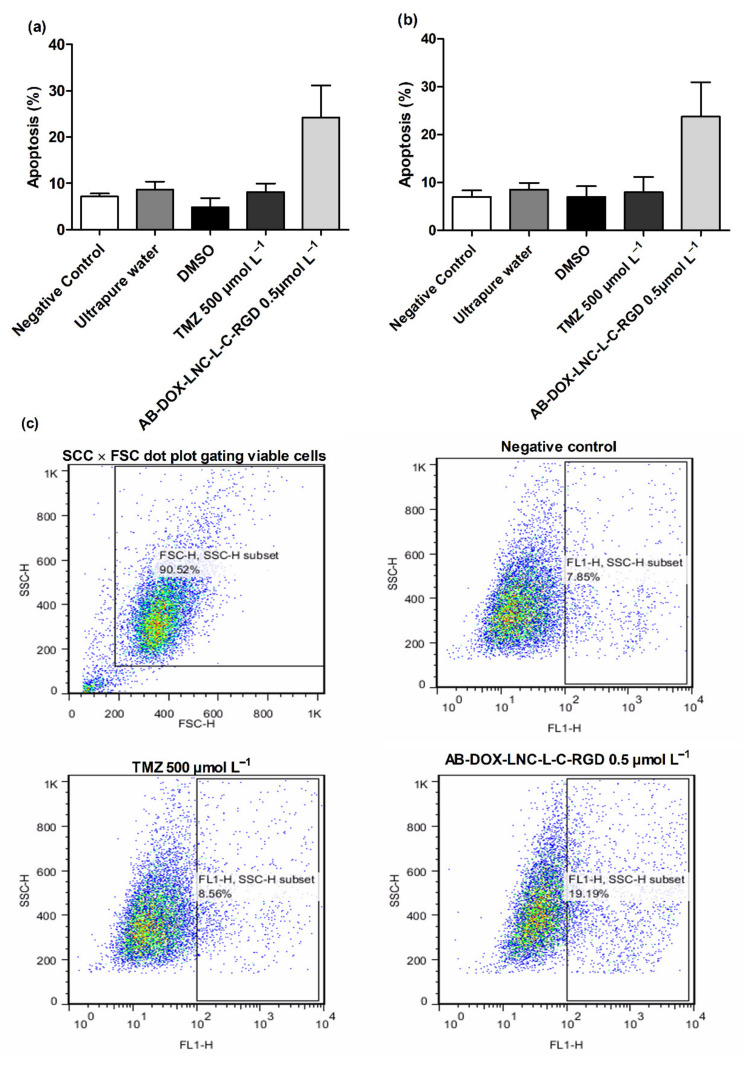
Apoptosis after 24 h of treatment for (**a**) U87MG cell line, (**b**) U138MG cell line, and (**c**) representative dot plots for U87MG cell line. Data are expressed as the mean ± SEM (*n* = 3). Negative control (untreated cells-control 1), ultrapure water (control 2), DMSO (control 3).

We additionally investigated apoptosis in the U87MG and U138MG cell lines using AnnexinV (Figure 4a,b); AB-DOX-LNC-L-C-RGD subtly increased apoptosis (24.23 ± 6.91%), while for the negative control, ultrapure water, and TMZ, the percentage of apoptotic cells detected was 7.14 ± 0.62, 8.69 ± 1.68, and 8.16 ± 1.79%, respectively, in the U87MG cell line (representative dot plots; Figure 4c). Similar results were observed in U138MG, where the proportions of apoptotic cells were 23.76 ± 7.20% with AB-DOX-LNC-L-C-RGD treatment, 6.98 ± 1.37% for the negative control, 8.52 ± 1.38% for ultrapure water, and 7.99 ± 3.15% for TMZ treatment (Figure 4b).

#### 3.3.3. Antiangiogenic Activity

**Figure 5:**
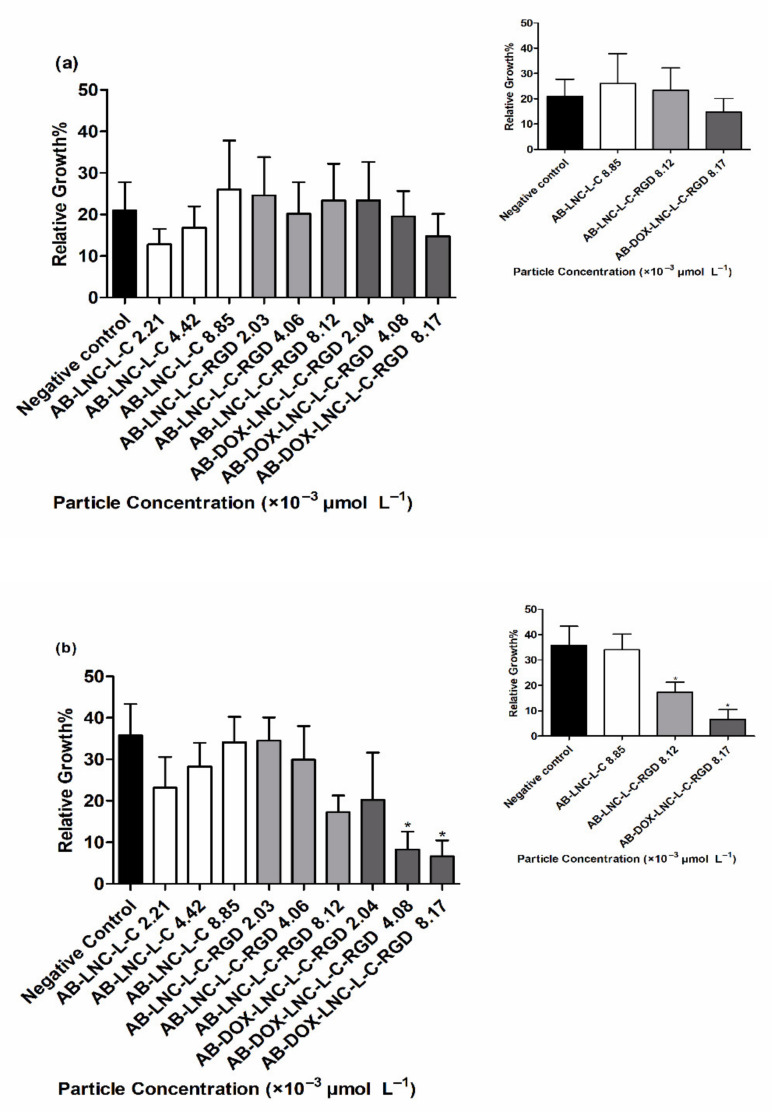
Relative vessel growth in chicken embryo CAM after (**a**) 24 h of treatment and (**b**) 48 h of treatment. Inset: Relative vessel growth following treatment at the highest concentrations of AB-LNC-L-C, AB-LNC-L-C-RGD, and AB-DOX-LNC-L-C-RGD. Data are expressed as the mean ± SEM. (*n* = 3) * represents the statistical difference with respect to the negative control (*p* < 0.05) (ANOVA, Dunnett).

Antiangiogenic activity was evaluated using a CAM assay after 24 h and 48 h of treatment. At 24 h, no differences were observed in RVG for all formulations and particle concentrations tested compared to that for the negative control (20.96 ± 6.80%) (Figure 5a) (*p* > 0.05). Treatment with AB-LNC-L-C-RGD (8.12 × 10^−3^ μmol L^−1^ particle concentration) resulted in a tendency to reduce angiogenesis in 48 h; however, the most promising results were observed with the formulation containing doxorubicin (AB-DOX-LNC-L-C-RGD) (Figure 5b).

The RVG after AB-DOX-LNC-L-C-RGD treatment at particle concentrations of 4.08 × 10^−3^ and 8.17 × 10^−3^ μmol L^−1^ was 8.26 ± 4.33 and 6.58 ± 3.90%, respectively, while the RVG in the negative control group was 35.77 ± 7.53% (Figure 5b). The reduction in vessel growth indicates the antiangiogenic effect of AB-DOX-LNC-L-C-RGD in a concentration-dependent manner; the highest applied concentration of AB-DOX-LNC-L-C-RGD (8.17 × 10^−3^ μmol L^−1^) resulted in 5.4 times less growth compared to the negative control (representative images of the CAM assay and raw data with the mean pixel values are shown in Supplementary Materials Figure S6 and Figure S7).

## 4. Discussion

Nanoformulation containing alpha-bisabolol and sorbitan monostearate, as core, and poly(ε-caprolactone), as shell, was prepared by self-assembling with adequate physicochemical characteristics. The nanocapsules were coated with chitosan by electrostatic interactions with lecithin, which is located between poly(ε-caprolactone) and chitosan. Polysorbate 80 was used to stabilize the colloids dispersed in water. On the surface of the nanoparticles, an organometallic complex was formed binding Zn^2+^ to the chitosan and RGD.

Doxorubicin, an inhibitor of DNA synthesis and topoisomerase [38,39] was combined with alpha-bisabolol, a membrane disruptor that induces pore formation in mitochondria and lysosomes, also responsible for caspase activation and cell death modulating the adenosynergic system in glioma cells [15,40]. RGD, a peptide that interacts with αvβ3 integrin expressed in GB cells and CAM endothelial cells, was used to promote active targeting.

The organometallic complexation is formed by a stable chelate between chitosan and the ligand, capable of being degraded in biological media [28]. In addition, the surface functionalization of the nanocapsules using the strategy described here did not cause aggregation; consequently, the size distribution curves (before and after surface functionalization) were almost superimposed. Our results are in agreement with previous studies [25,27], in which no significant difference was observed in the z-average diameters before and after surface functionalization with phenylalanine or laronidase, respectively. Nevertheless, in other reports, functionalization of nanocapsules with anti-LDL(−)-single-chain variable antibody fragment [25,26] or bevacizumab [28] resulted in increases of approximately 60 nm or 20 nm in the z-average diameters, respectively. Notably, small molecules such as phenylalanine and RGD might not influence the hydrodynamic mean diameter after complexation on the nanocapsule surface, whereas molecules presenting higher molecular weights such as a single-chain variable antibody fragment or an antibody can slightly affect the size distribution, leading to an increase of a few nanometers in the mean diameter.

Furthermore, the diameters obtained by DLS and NTA were slightly different for all formulations evaluated. Although the two techniques are based on the Brownian motion of the particles, the difference can be related to the method of evaluation of the particle size, because DLS measures fluctuations in the intensity of light scattering caused by the translation of millions of particles, while NTA records the random motion of hundreds of particles individually. We also observed a reduction in PND for AB-DOX-LNC-L-C-RGD, which is a consequence of the dilutions caused by the coating and surface functionalization steps in the synthesis.

The zeta potential of the AB-LNC-L and AB-DOX-LNC-L formulation was negative because of the presence of phosphate groups (PO_4_^−^) in phosphatidic acid (lecithin contaminant). The zeta potential value and the lack of microaggregates in the size distribution curves indicate that the mechanism of the kinetic stability of the formulation is primarily based on steric hindrance instead of electrostatic repulsion. Besides, we demonstrated that polysorbate 80 forms micelles at the particle–water interface, contributing to the formulation’s kinetic stability by steric hindrance. Electrostatic repulsion and steric hindrance are responsible for nanoparticle stability [41,42,43]. After coating with chitosan, the zeta potential was inverted to positive values due to the polycationic nature of chitosan [41,44]. We recently demonstrated that the presence of chitosan at the nanocapsule surface is essential for promoting metal binding [28], favoring organometallic complexation with different ligands [20,25,27,28]. RGD-surface-functionalized nanocapsules maintained positive zeta potential values. However, in nanocapsules containing alpha-bisabolol (without doxorubicin), the zeta potential value varied from +18.4 ± 0.4 for AB-LNC-L-C to +14.9 ± 1.2 for AB-LNC-L-C-RGD. This reduction suggests that the carboxylic acid groups of aspartic acid are ionized (negative charge) and the arginine portion complexed with the metal.

Formulations containing chitosan as the coating material exhibited acid pH values in accordance with previously studies [45,46]. Furthermore, the chitosan coating promoted a reduction in the doxorubicin encapsulation efficiency. Doxorubicin is an ionizable molecule, so the addition of the chitosan solution led to the ionization of amine groups of doxorubicin, displacing the drug for external regions of particles and/or continuous phase. The reduction in doxorubicin content after coating and surface functionalization are related to successive dilutions for the synthesis process as well as observed for the concentration of particles.

The drug content and encapsulation efficiency of alpha-bisabolol as well as RGD are in agreement with the values previously observed for alpha-bisabolol-loaded-LNC [37], a formulation prepared without using doxorubicin or RGD, and for RGD-surface-functionalized DOX-loaded-LNC [29], a formulation prepared without alpha-bisabolol.

In a previous study, we demonstrated that RGD-surface-functionalized drug-unloaded LNC, when applied in U78MG cells, reduced cell viability after 24 h of exposure. In addition, RGD-surface-functionalized doxorubicin-loaded LNC (prepared without alpha-bisabolol) showed higher cytotoxicity compared to doxorubicin hydrochloride solution at all evaluated concentrations (1.7–17 μmol mL^−1^, at particle concentrations from 1.03 × 10^−4^ to 2.06 × 10^−4^ μmol L^−1^) [29]. In another study, we verified a greater antitumoral effect (GB cells) of alpha-bisabolol loaded-LNC compared to alpha-bisabolol in solution (data not shown).

In the present study, nanoformulation containing alpha-bisabolol, doxorubicin, and RGD were used at concentrations from 1.56 × 10^−6^ to 1.57 × 10^−4^ μmol L^−1^ (nanocapsules) and 0.01 to 1 μmol L^−1^ (doxorubicin). A direct correlation was observed between dose of doxorubicin and its cellular internalization. The results corroborate the reduction in cell viability observed in GB cells by the MTT assay. In addition, the treatment with AB-DOX-LNC-L-C-RGD exhibited superior cytotoxic activity compared with TMZ. Our findings are in agreement with the study of Lundy et al., which demonstrated that liposomes containing doxorubicin reduced the viability of GB in vitro more than TMZ (human GB cell line DBTRG-05MG) [47]. Interestingly, AB-DOX-LNC-L-C-RGD showed promising cytotoxic effects in the U138MG cell line, which has been reported to be resistant to TMZ treatment [48,49].

Several studies have used RGD-surface-functionalized nanocarriers and doxorubicin encapsulation as a strategy against GB. In this context, Song et al. developed RGD-functionalized nanoparticles containing doxorubicin and TNP-470, an endothelial inhibitor. The cytotoxic potential was assessed in U87MG cells [13]. The median inhibition cell viability was observed at 6.7 μg mL^−1^ doxorubicin-equivalent concentration after 48 h of treatment. Similarly, Belhadj et al. observed that the IC_50_ of doxorubicin-loaded liposomes functionalized with cyclic RGD and p-hydroxybenzoic acid was 4.79 μmol L^−1^ (doxorubicin-equivalent; 72 h incubation) [50]. Other studies have demonstrated the cytotoxic effect of doxorubicin-loaded nanoparticles functionalized with different surface ligands in U87MG [51,52]; however, higher doxorubicin-equivalent concentrations were used in these studies compared to that in the present study [IC_50_ close to 0.5 μmol L^−1^ (0.3 μg mL^−1^)].

However, we also observed toxicity of AB-DOX-LNC-L-C-RGD in HaCaT, MRC-5, and HepG2 cells lines. Despite the use of HepG2 in models for assessing hepatotoxicity, this is a tumor cell line that is susceptible to doxorubicin [53,54] and alpha-bisabolol [55]. In contrast, the HaCaT cell line has a high level of topoisomerase I activity; therefore, it is sensitive to topoisomerase inhibitors such as doxorubicin [56]. Additionally, in vitro cytotoxicity of doxorubicin has been observed in MRC-5 [54]. In this study, the HaCaT, MRC-5, and HepG2 cell lines were more sensitive to treatment with AB-DOX-LNC-L-C-RGD and TMZ than GB cells. The in vitro toxicity assay has some limitations when applied to study nanoparticle systems. Studies have demonstrated that toxicity may be higher in the monolayer cell culture (2D) than in more complex in vitro assays such as 3D cell culture [12,57] and in vivo assays [58]. Besides, in in vitro monoculture cells, there is no interaction with other cell types and no physiological and mechanical stimuli (such as blood flow) [59].

In the cell cycle assay, our findings corroborate previous studies in the literature, which demonstrated that TMZ-induced G2/M cell cycle arrest [60,61]. Additionally, a previous study reported S-phase arrest following 24 h of treatment with unencapsulated doxorubicin, with a subsequent increase in the proportion of cells in the G2 phase (48 h) [62]. S-phase arrest was also observed with RGD peptide-decorated and doxorubicin-loaded selenium nanoparticles (RGD-NPs) in HUVEC cells [63]; in contrast, in other studies, alpha-bisabolol and peptides containing the RGD sequence (dimeric RGD sequence) alone increased the proportion of cells in the G1 phase [64,65]. Doxorubicin is a topoisomerase inhibitor and a known DNA synthesis inhibitor because of its ability to intercalate between the nitrogenous bases, alter DNA conformation, and induce damage [38,39]. Considering the importance of DNA synthesis in cell cycle progression, these actions of doxorubicin can be the major factors involved in the S-phase arrest observed after the application of nanocapsules in the U87MG and U138MG cell lines. In addition, apoptosis is a cell death mechanism induced by doxorubicin through p53 activation and alterations in the BCL-2/Bax ratio [38]. However, doxorubicin has also been reported to induce necrosis [54,62]. Furthermore, it has been described that alpha-bisabolol can promote cytochrome C release from the mitochondria to the cytosol, caspase-3 activation, and reduction in the ratio of BCL-2/Bax, and pro-apoptotic pathways in several cancer cell lines [14,15,65].

To assess the antiangiogenic potential of the formulation, we used the CAM assay. The CAM is an extraembryonic membrane, initially poorly vascularized, but during incubation period and development of the embryo, provides a rich vascular plexus formed due to the angiogenesis process [33]. The main period of growth of the vessels is until day 11 of incubation [66]. The exposition of the CAM to substances with antiangiogenic activity leads to a reduction in the normal vessels’ development. In this way, the antiangiogenic effect observed is related to the interference in the formation and growth of vessels in the CAM.

The vascular endothelium of CAM exhibits a high expression of integrin αvβ3 to which RGD can bind [67]. Different studies have reported angiogenesis inhibition when RGD-peptides (cyclic RGD and ST1646 RGD peptide) or antibody antagonists of αvβ3 integrin were administered in CAM [68,69].

In this study, we developed a RGD-surface functionalized nanoparticle. The presence of RGD on the particle surface favors recognition by αvβ3 integrin on the surface of endothelial cells. This recognition may be favoring the targeting of the particle and delivery of alpha-bisabolol and doxorubicin to the cells. Furthermore, the experiment was made within the sixth to eighth days of incubation the period in which CAM endothelial cells are in a constant proliferation process [66]. This fact can favor the action of doxorubicin, since its mechanisms of action are the intercalation of DNA and inhibition of topoisomerase [38]. In addition, doxorubicin was reported to induce cytotoxicity and disrupt tube-like structures formed by human umbilical vein endothelial cells (HUVECs) as well as reduce migration, cell proliferation, and angiogenesis, according to the CAM assay [70]. Additionally, Magnelli et al. described the antiangiogenic properties of alpha-bisabolol through the induction of apoptosis and inhibition of the formation of tube-like structures by HUVECs cells in vitro [71].

In summary, the combination of the three active substances in the AB-DOX-LNC-L-C-RGD nanocapsule exhibited a more effective antiangiogenic activity compared to controls; however, doxorubicin plays an important role in the efficacy of the nanocarrier. Despite the antiangiogenic effect and the cytotoxicity observed in vitro in non-tumor cells, the embryos remained viable during the 48 h of exposure to AB-DOX-LNC-L-C-RGD.

## 5. Conclusions

In this study, we demonstrated that the nanoformulation AB-DOX-LNC-L-C-RGD reduced the viability of GB cells through programmed cell death and promoted S-phase cell cycle arrest. These effects were observed even for GB cells resistant to conventional chemotherapy with TMZ. In addition, AB-DOX-LNC-L-C-RGD is capable of inhibiting angiogenesis, as shown in the CAM model, permitting us to envisage its application for the treatment of a variety of solid tumors. Collectively, the results support the therapeutic potential of the nanoformulation against GB and, thereby, pave the way for future preclinical studies.

## Figures and Tables

**Table 1:** Physicochemical properties of the formulations.

Parameters	AB-LNC-L	AB-LNC-L-C	AB-LNC-L-C-RGD	AB-DOX-LNC-L	AB-DOX-LNC-L-C	AB-DOX-LNC-L-C-RGD
d*h* z-ave (nm)	139 ± 10	146 ± 6	141 ± 10	140 ± 1	147 ± 4	138 ± 2
PDI	0.12 ± 0.01	0.17 ± 0.00	0.14 ± 0.01	0.15 ± 0.03	0.18 ± 0.00	0.19 ± 0.01
D*h* (nm)	136 ± 16	154 ± 2	148 ± 10	126 ± 5	141 ± 8	170 ± 19
PND (part. mL^−1^)	(2.09 ± 0.95) × 10^13^	(1.59 ± 0.31) × 10^13^	(4.89 ± 1.5) × 10^12^	(2.44 ± 0.4) × 10^13^	(1.31 ± 0.14) × 10^13^	(4.90 ± 0.8) × 10^12^
ZP (mV)	−11.4 ± 0.6	+18.4 ± 0.4	+14.9 ± 1.2	−11.6 ± 1.5	+11.8 ± 2.0	+13.4 ± 1.0
pH	5.79 ± 0.38 ^1)^	4.04 ± 0.01 ^1)^	4 ^2)^	7.30 ± 0.3 ^1)^	4.18 ± 0.04 ^1)^	4 ^2)^

z-average hydrodynamic mean diameter (d*h* z-ave) and polydispersity index (PDI) determined by dynamic light scattering, hydrodynamic mean diameter by number of particles (D*h*), and particle number density (PND) determined by nanoparticle tracking analysis, zeta potential (ZP) determined by electrophoretic light scattering, pH determined by potentiometry ^1)^ or by pH indicator strips ^2)^. Data were expressed as mean ± SD (*n* = 3).

**Table 2:** Doxorubicin content determined by liquid chromatography and encapsulation efficiency of doxorubicin (EE%) determined by ultrafiltration-centrifugation following quantification.

Parameters	AB-DOX-LNC-L	AB-DOX-LNC-L-C	AB-DOX-LNC-L-C-RGD
Doxorubicin content (µg mL**^−1^**)	94.88 ± 2.61	85.39 ± 0.82	30.79 ± 0.53
EE%	98.07 ± 0.7	64.32 ± 7.15	65.89 ± 3.63

Data were expressed as mean ± SD (*n* = 3).

## Data Availability

All data generated or analyzed during this study are included in this published article and its supplementary information files.

## Supplementary Materials

The following are available online at https://www.mdpi.com/article/10.3390/pharmaceutics13060862/s1, Figure S1: Size distribution curves of different batches of formulations by dynamic light scattering analysis, Table S1: MTT assay, Figure S2: Doxorubicin cell uptake after 6 h of treatment with different concentrations of AB-DOX-LNC-L-C-RGD in glioblastoma cells; Figure S3: Optical microscopy images of U87MG cancer cells after 24 h of treatment, Figure S4: Optical microscopy images of U138MG cancer cells after 24 h of treatment, Figure S5: Cell viability by MTT assay after 24 h of treatment, Table S2: CAM Assay, Figure S6: Representative images of CAM assay, Figure S7: Raw data of CAM assay in pixel mean value at t0, t24, and, t48 h of treatment.
