# Supplementary material for: Nanoformulation Shows Cytotoxicity against Glioblastoma Cell Lines and Antiangiogenic Activity in Chicken Chorioallantoic Membrane
Source: Pharmaceutics. 2021 Jun 11;13(6):862. doi: 10.3390/pharmaceutics13060862 (PMC8230781; doi:10.3390/pharmaceutics13060862)
Supplement: Supplementary file 1 [file pharmaceutics-13-00862-s001.zip › pharmaceutics-1230430-supplementary.pdf]

pharmaceutics

MDPI

Article

Supplementary materials: Nanoformulation Shows Cytotoxicity against Glioblastoma Cell Lines and Antiangiogenic Activity in Chicken Chorioallantoic Membrane

Danieli Rosane Dallemole, Thatiana Terroso, Aline de Cristo Soares Alves, Juliete Nathali Scholl, Giovana Ravizzoni Onzi, Rodrigo Cé, Karina Paese, Ana Maria Oliveira Battastini, Silvia Stanisçuaski Guterres; Fabrício Figueiró; Adriana Raffin Pohlmann

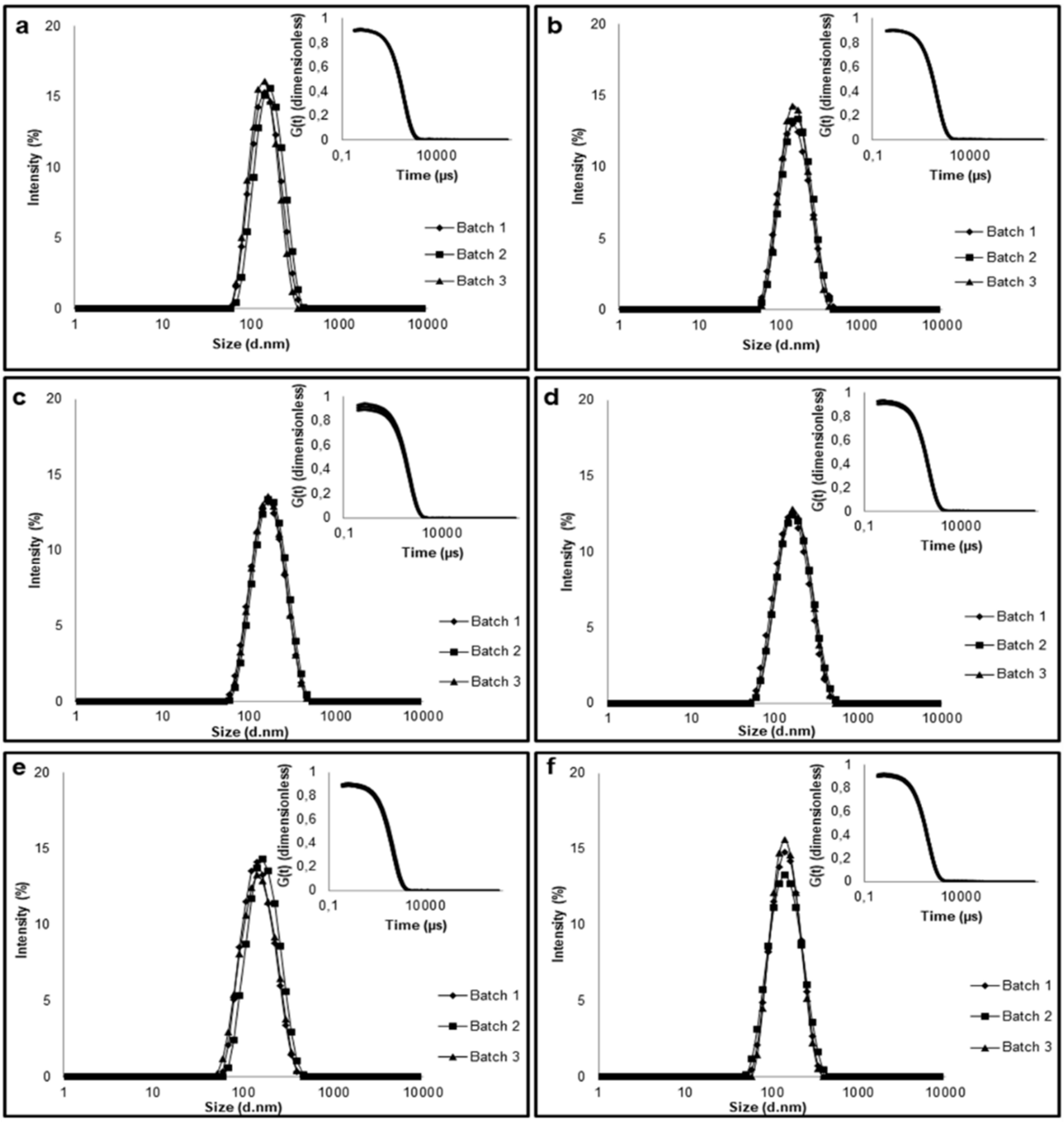

Figure S1. Size distribution curves of different batches of formulations by dynamic light scattering analysis. Inset: correlation function. (**a**) AB-LNC-L, (**b**) AB-DOX-LNC-L, (**c**) AB-LNC-L-C, (**d**) AB-DOX-LNC-L-C, (**e**) AB-LNC-L-C-RGD, (**f**) AB-DOX-LNC-L-C-RGD.

Table S1: MTT assay. Concentration of nanocapsules (particle concentration), concentration of alpha-bisabolol, concentration of doxorubicin, and concentration of arginylglycylaspartic acid in AB-DOX-LNC-L-C-RGD formulation studied in MTT assay.

Treatment	Part. Conc. (µmol L^{-1})	AB. Conc. (µmol L^{-1})	DOX. Conc. (µmol L^{-1})	RGD Conc. (µmol L^{-1})
AB-DOX-LNC-L-C-RGD 0.01	1.56×10^{-6}	7.09	0.01	0.146
AB-DOX-LNC-L-C-RGD 0.05	7.78×10^{-6}	35.44	0.05	0.734
AB-DOX-LNC-L-C-RGD 0.1	1.56×10^{-5}	70.88	0.10	1.460
AB-DOX-LNC-L-C-RGD 0.5	7.79×10^{-5}	354.42	0.50	7.340
AB-DOX-LNC-L-C-RGD 1.0	1.57×10^{-4}	712.86	1.00	14.770

Part. Conc.: Particle concentration, AB: Alpha-bisabolol, DOX: Doxorubicin, RGD: Arginylglycylaspartic

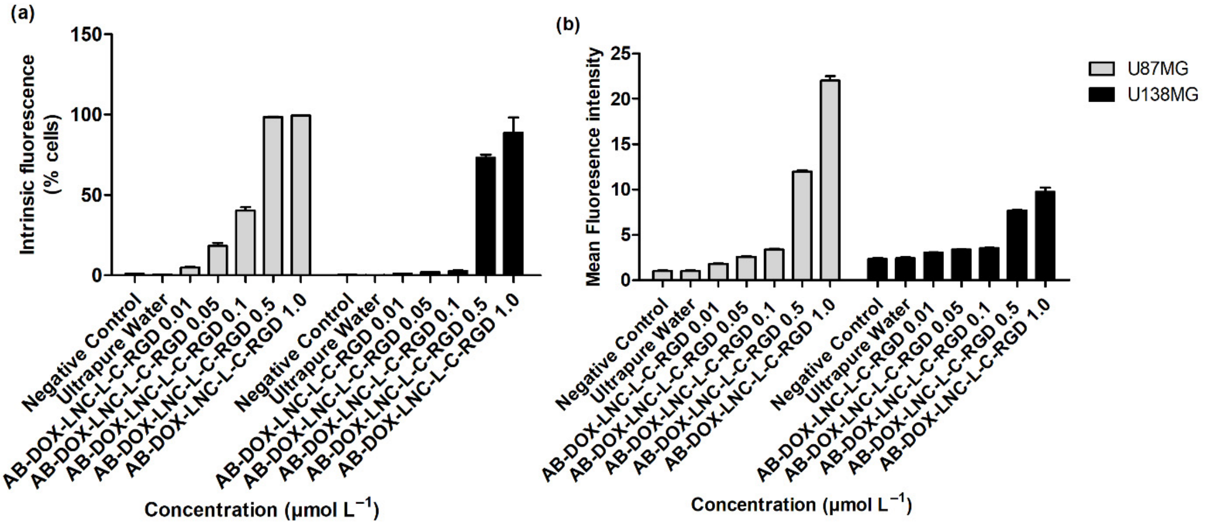

Figure S2. Doxorubicin cell uptake after 6 h of treatment with different concentrations of AB-DOX-LNC-L-C-RGD in glioblastoma cells: (**a**) percentage of cells considered as doxorubicin-positive (intrinsic florescence % cells) for U87MG and U138MG and (**b**) mean fluorescence intensity for U87MG and U138MG. Fluorescence was analyzed in the FL3 channel. Negative control (untreated cells-control 1), ultrapure water (control 2).

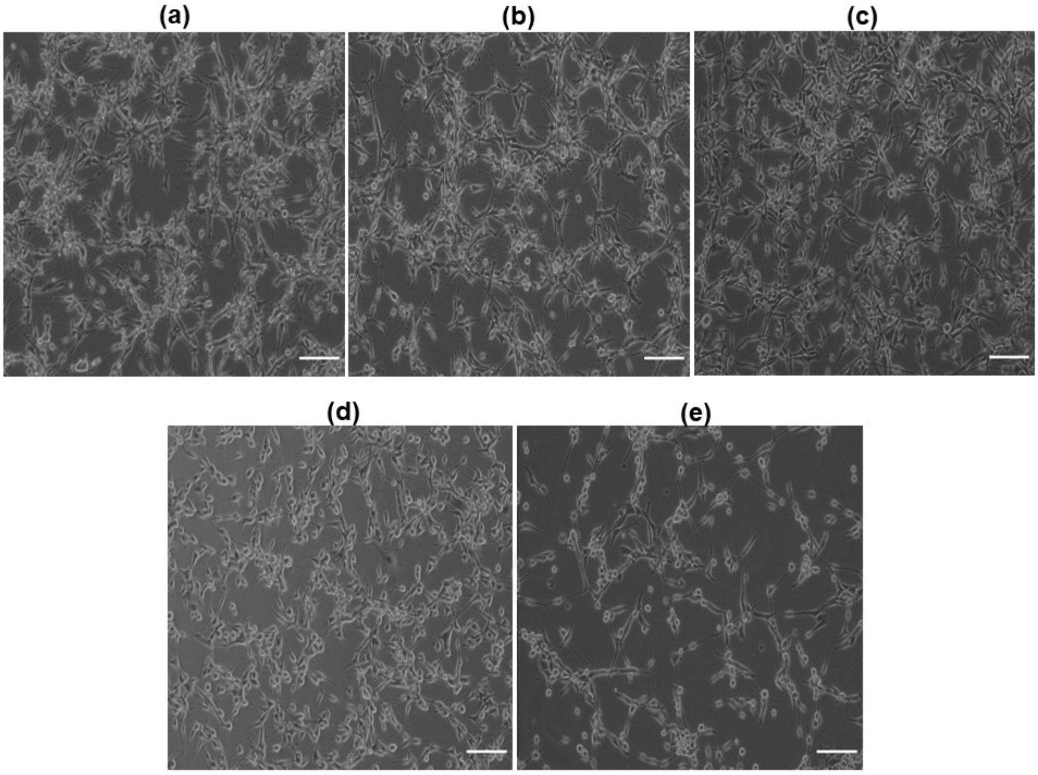

Figure S3. Optical microscopy images of U87MG cancer cells after 24 h of treatment: (**a**) Negative control (untreated cells - control 1), (**b**) Ultrapure water (control 2), (**c**) DMSO (control 3), (**d**) TMZ at 500 µmol L^{-1}, (**e**) AB-DOX-LNC-L-C-RGD at 0.5 µmol L^{-1} of doxorubicin. Scale Bar = 100µm.

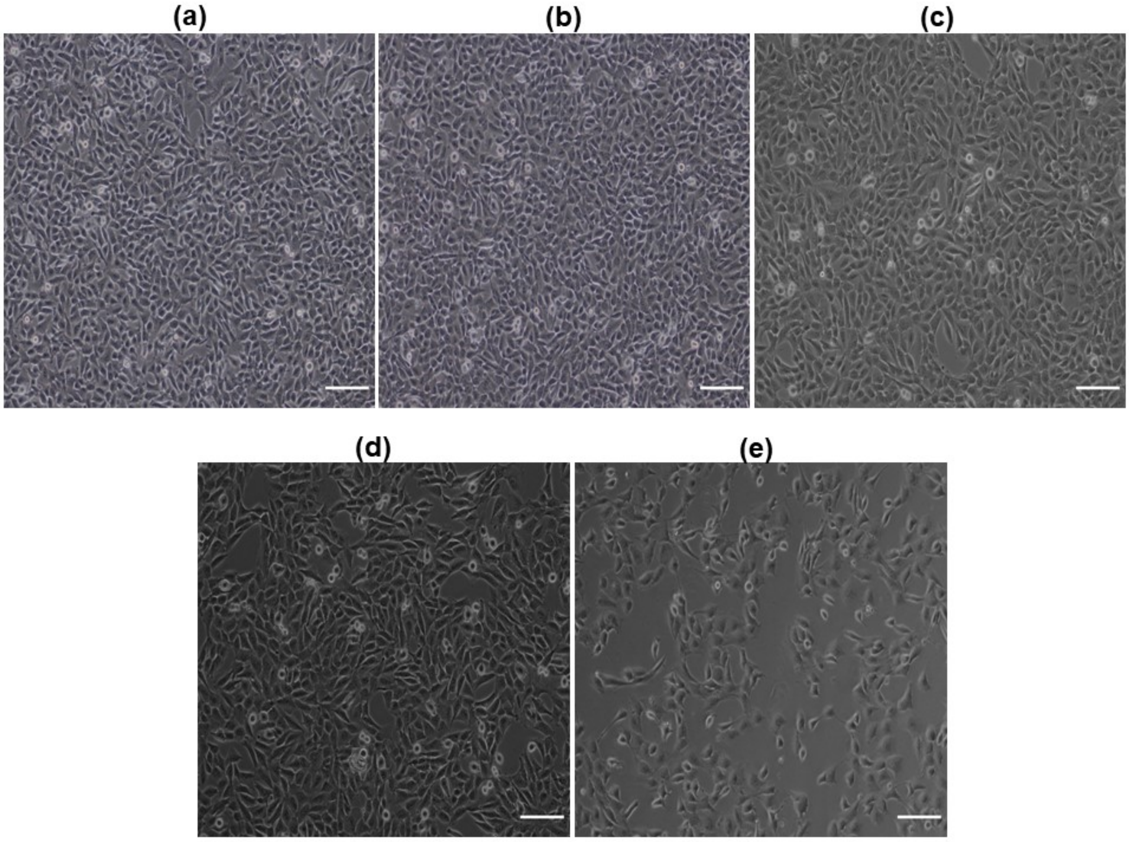

Figure S4. Optical microscopy images of U138MG cancer cells after 24 h of treatment: (**a**) Negative control (untreated cells - control 1), (**b**) Ultrapure water (control 2), (**c**) DMSO (control 3), (**d**) TMZ at 500 µmol L^{-1}, (**e**) AB-DOX-LNC-L-C-RGD at 0.5 µmol L^{-1} of doxorubicin. Scale Bar = 100µm.

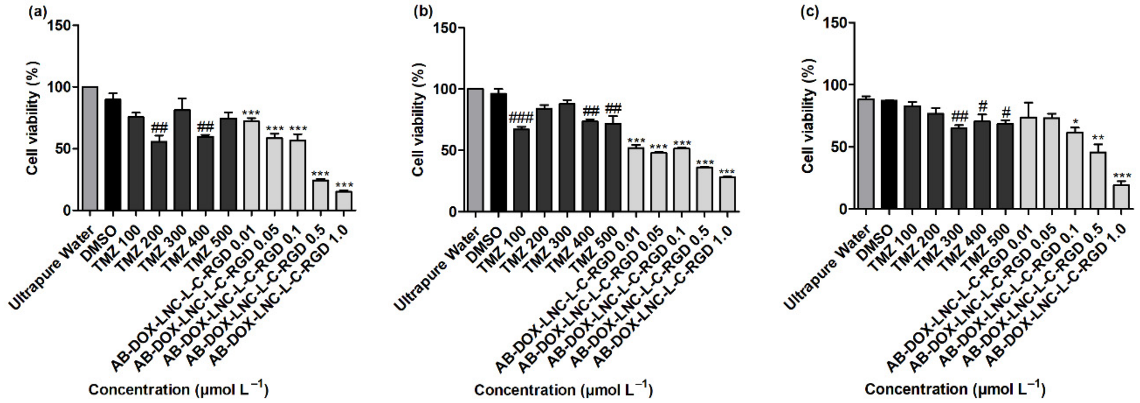

Figure S5. Cell viability by MTT assay after 24 h of treatment: (**a**) HaCaT cell line, (**b**) MRC-5 cell line and (**c**) HepG2 cell line. Ultrapure water (control 2), DMSO (control 3) * represent statistical difference from the control (ultrapure water) ($p < 0.05$). ** represent statistical difference from the control (ultrapure water) ($p < 0.01$). *** represent statistical difference from the control (ultrapure water) ($p < 0.001$). # represent statistical difference from the control (DMSO) ($p < 0.05$). ## represent statistical difference from the control (DMSO) ($p < 0.01$). ### represent statistical difference from the control (DMSO) ($p < 0.001$) (ANOVA, Dunnett).

Table S2 CAM Assay. Concentration of nanocapsules (particle concentration), concentration of alpha-bisabolol, concentration of doxorubicin, and concentration of arginylglycylaspartic acid in the formulations studied in CAM assay (volume of sample applied = 100 μL).

Treatment	Part. Conc. (μmol L^{-1})	AB. Conc. (μmol L^{-1})	DOX. Conc. (μmol L^{-1})	RGD Conc. (μmol L^{-1})
Negative control (0.9% NaCl solution)	-	-	-	-
AB-LNC-L-C (2.21 × 10^{-3} μmol L^{-1})	2.21 × 10^{-3}	10068.70	-	-
AB-LNC-L-C (4.42 × 10^{-3} μmol L^{-1})	4.42 × 10^{-3}	20137.39	-	-
AB-LNC-L-C (8.85 × 10^{-3} μmol L^{-1})	8.85 × 10^{-3}	40274.78	-	-
AB-LNC-L-C-RGD (2.03 × 10^{-3} μmol L^{-1})	2.03 × 10^{-3}	10068.70	-	208.60
AB-LNC-L-C-RGD (4.06 × 10^{-3} μmol L^{-1})	4.06 × 10^{-3}	20137.39	-	416.20
AB-LNC-L-C-RGD (8.12 × 10^{-3} μmol L^{-1})	8.12 × 10^{-3}	40274.78	-	834.40
AB-DOX-LNC-L-C-RGD (2.04 × 10^{-3} μmol L^{-1})	2.04 × 10^{-3}	10068.70	14.16	208.60
AB-DOX-LNC-L-C-RGD (4.08 × 10^{-3} μmol L^{-1})	4.08 × 10^{-3}	20137.39	28.32	416.20
AB-DOX-LNC-L-C-RGD (8.17 × 10^{-3} μmol L^{-1})	8.17 × 10^{-3}	40274.78	56.65	834.40

Part. Conc.: Particle concentration; AB: Alpha-bisabolol; DOX: Doxorubicin; RGD: Arginylglycylaspartic acid.

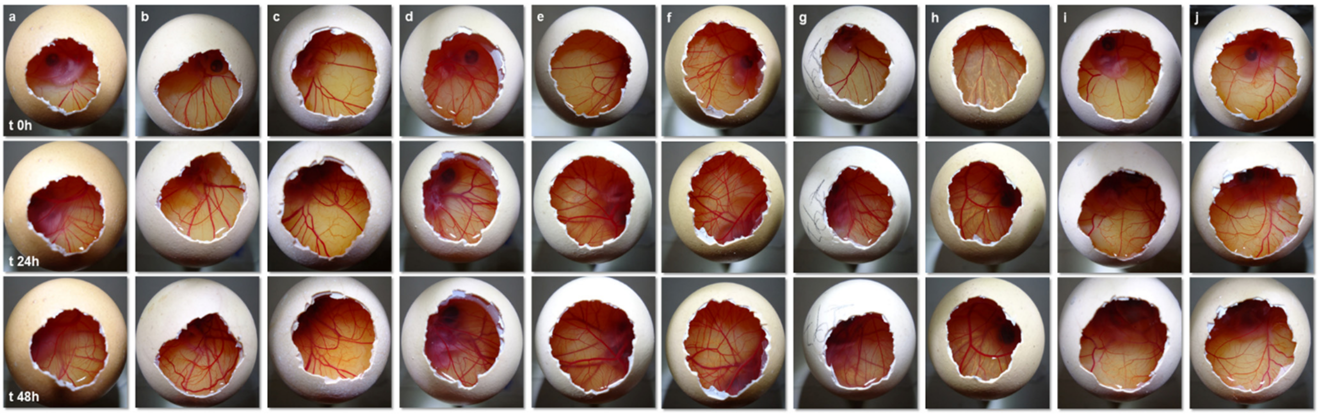

Figure S6. Representative images of CAM assay: (**a**) Negative control; (**b**) AB-LNC-L-C (2.21×10^{-3} μmol L^{-1}); (**c**) AB-LNC-L-C (4.42 × 10^{-3} μmol L^{-1}); (**d**) AB-LNC-L-C (8.85 × 10^{-3} μmol L^{-1}); (**e**) AB-LNC-L-C-RGD (2.03 × 10^{-3} μmol L^{-1}); (**f**) AB-LNC-L-C-RGD (4.06 × 10^{-3} μmol L^{-1}); (**g**) AB-LNC-L-C-RGD (8.12 × 10^{-3} μmol L^{-1}); (**h**) AB-DOX-LNC-L-C-RGD (2.04 × 10^{-3} μmol L^{-1}); (**i**) AB-DOX-LNC-L-C-RGD (4.08 × 10^{-3} μmol L^{-1}); (**j**) AB-DOX-LNC-L-C-RGD (8.17 × 10^{-3} μmol L^{-1}). The value described in parentheses refers to the concentration of particles applied in the CAM. Each column represents a different group, while the lines represent different treatment time intervals.

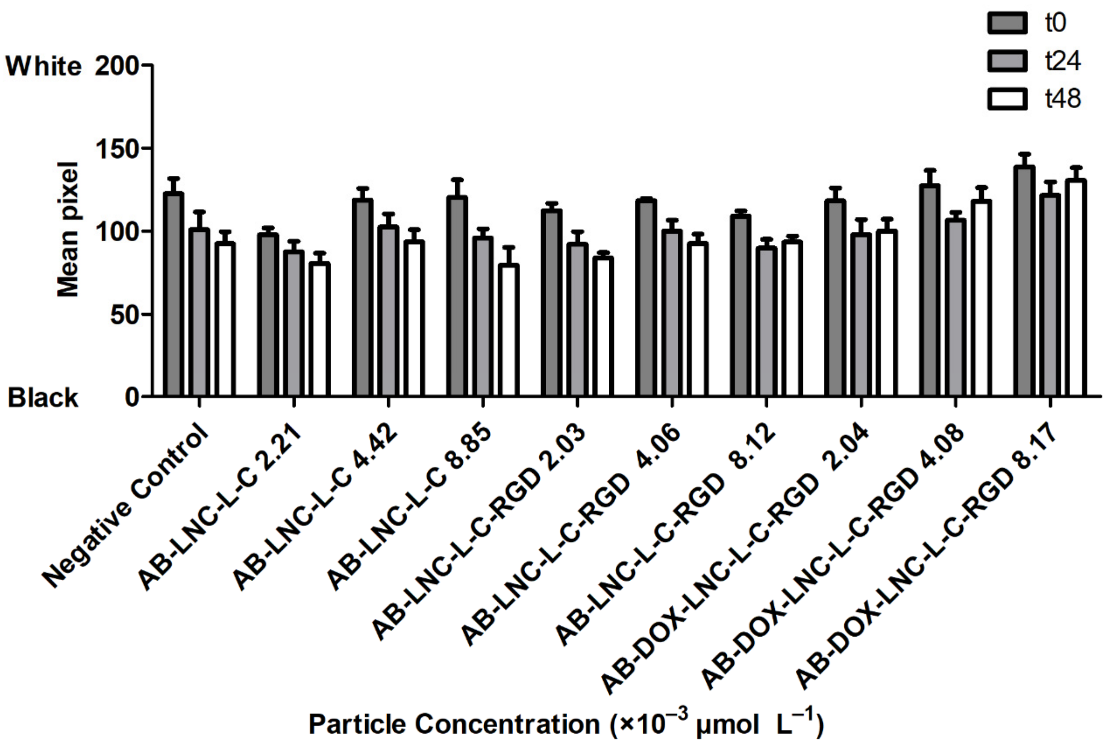

Figure S7. Raw data of CAM assay in pixel mean value at t0, t24 and, t48 h of treatment. The pixel scale ranges from 0 to 255, with 0 referring to black and 255 to white. The mean pixel of the grayscale photo is closer to 0 when the angiogenesis is increased, and the mean pixel value is closer to 255 when the angiogenesis is decreased (inhibition of vessels growth).
